# Genetic diversity of vector-borne pathogens in spotted and brown hyenas from Namibia and Tanzania relates to ecological conditions rather than host taxonomy

**DOI:** 10.1186/s13071-021-04835-x

**Published:** 2021-06-16

**Authors:** Jürgen Krücken, Gábor Á. Czirják, Sabrina Ramünke, Maria Serocki, Sonja K. Heinrich, Jörg Melzheimer, M. Carolina Costa, Heribert Hofer, Ortwin H. K. Aschenborn, Nancy A. Barker, Stefano Capodanno, Luís Madeira de Carvalho, Georg von Samson-Himmelstjerna, Marion L. East, Bettina Wachter

**Affiliations:** 1 Institute for Parasitology and Tropical Veterinary Medicine, Freie Universität Berlin, Berlin, Germany; 2 Department of Wildlife Diseases, Leibniz Institute for Zoo and Wildlife Research, Berlin, Germany; 3 Department of Evolutionary Ecology, Leibniz Institute for Zoo and Wildlife Research, Berlin, Germany; 4 Centro de Investigação Interdisciplinar Em Sanidade Animal, Faculdade de Medicina Veterinária, Universidade de Lisboa, Lisbon, Portugal; 5 Leibniz Institute for Zoo and Wildlife Research, Berlin, Germany; 6 Department of Veterinary Medicine, Freie Universität Berlin, Berlin, Germany; 7 Department of Biology, Freie Universität Berlin, Berlin, Germany; 8 School of Veterinary Medicine, University of Namibia, Windhoek, Namibia; 9 School of Life Sciences, University of KwaZulu-Natal, Durban, South Africa; 10 Department of Veterinary Medicine, University Federico II of Naples, Naples, Italy; 11 Department of Ecological Dynamics, Leibniz Institute for Zoo and Wildlife Research, Berlin, Germany

**Keywords:** Wildlife parasites, Vector-borne diseases, Tick-borne diseases, Carnivores, Hyenas, Pathogen ecology

## Abstract

**Background:**

Improved knowledge on vector-borne pathogens in wildlife will help determine their effect on host species at the population and individual level and whether these are affected by anthropogenic factors such as global climate change and landscape changes. Here, samples from brown hyenas (*Parahyaena brunnea*) from Namibia (BHNA) and spotted hyenas (*Crocuta crocuta*) from Namibia (SHNA) and Tanzania (SHTZ) were screened for vector-borne pathogens to assess the frequency and genetic diversity of pathogens and the effect of ecological conditions and host taxonomy on this diversity.

**Methods:**

Tissue samples from BHNA (*n* = 17), SHNA (*n* = 19) and SHTZ (*n* = 25) were analysed by PCRs targeting Anaplasmataceae, *Rickettsia* spp., piroplasms, specifically *Babesia lengau*-like piroplasms, Hepatozoidae and filarioids. After sequencing, maximum-likelihood phylogenetic analyses were conducted.

**Results:**

The relative frequency of Anaplasmataceae was significantly higher in BHNA (82.4%) and SHNA (100.0%) than in SHTZ (32.0%). Only *Anaplasma phagocytophilum*/*platys*-like and *Anaplasma bovis*-like sequences were detected. *Rickettsia raoultii* was found in one BHNA and three SHTZ. This is the first report of *R. raoultii* from sub-Saharan Africa. *Babesia lengau*-like piroplasms were found in 70.6% of BHNA, 88.9% of SHNA and 32.0% of SHTZ, showing higher sequence diversity than *B. lengau* from South African cheetahs (*Acinonyx jubatus*). In one SHTZ, a *Babesia vogeli*-like sequence was identified. *Hepatozoon felis*-like parasites were identified in 64.7% of BHNA, 36.8% of SHNA and 44.0% of SHTZ. Phylogenetic analysis placed the sequences outside the major *H. felis* cluster originating from wild and domestic felids. Filarioids were detected in 47.1% of BHNA, 47.4% of SHNA and 36.0% of SHTZ. Phylogenetic analysis revealed high genetic diversity and suggested the presence of several undescribed species. Co-infections were frequently detected in SHNA and BHNA (BHNA median 3 pathogens, range 1–4; SHNA median 3 pathogens, range 2–4) and significantly rarer in SHTZ (median 1, range 0–4, 9 individuals uninfected).

**Conclusions:**

The frequencies of all pathogens groups were high, and except for *Rickettsia*, multiple species and genotypes were identified for each pathogen group. Ecological conditions explained pathogen identity and diversity better than host taxonomy.

**Graphic Abstract:**

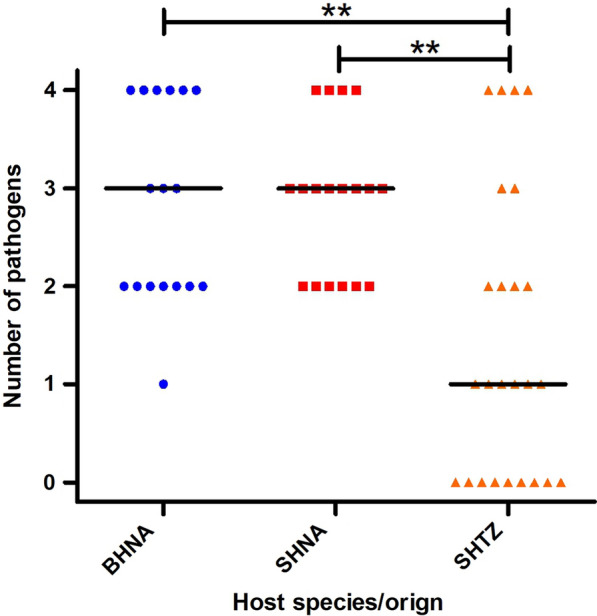

**Supplementary Information:**

The online version contains supplementary material available at 10.1186/s13071-021-04835-x.

## Background

Vector-borne pathogens can cause severe diseases in carnivores, such as leishmaniasis [1], babesiosis [2, 3] or cardiopulmonary dirofilariasis [4, 5]. Whereas canine vector-borne diseases have received much attention during the past decades, feline diseases were less often investigated [6–9]. Even less investigated are pathogens of wild carnivores, which are often only addressed if wild carnivore populations are important reservoirs of either severe diseases of closely related domestic animals or zoonotic pathogens. For example, *Babesia rossi* has its natural reservoir in wild canids such as side-striped jackals (*Canis adustus*) and African wild dogs (*Lycaon pictus*) and causes hyper-virulent babesiosis in domestic dogs in sub-Saharan Africa [10]. Important zoonotic vector-borne pathogens of carnivores with reservoirs in wildlife are *Leishmania infantum*, *Dirofilaria immitis* and *Dirofilaria repens* [11]. Studies on vector-borne pathogens of wild carnivores focus on temperate and Mediterranean European regions, the United States and South Africa, so current knowledge on vector-borne pathogens in other tropical and subtropical regions and their impact on humans, domestic species and wildlife is limited [12–14]. Furthermore, increased contact between domestic animals and wildlife associated with human degradation of natural habitats can pose a health threat to wildlife populations, since domestic animals might serve as reservoirs for and amplifiers of vector-borne pathogens transmitted to wildlife [15].

Our study aimed to use molecular techniques to determine the presence, identity and diversity of vector-borne pathogens in two species in the family Hyaenidae to investigate the importance of host species and sampling location and habitat [16] with respect to infection. For this purpose, we collected blood and tissue samples from brown hyenas (*Parahyaena brunnea* Thunberg 1820) in Namibia and from spotted hyenas (*Crocuta crocuta* Erxleben 1777) in Namibia (Southern Africa) and Tanzania (East Africa) from three major habitats which vary in ecological characteristics and the kind of and the degree to which human activities affect vector and host populations. Namibian livestock farmland rarely burns, and contains livestock, companion animals, artificial water sources and possibly cattle dips to control ticks. Etosha National Park is not regularly burnt and has artificial waterholes. In the Serengeti National Park in Tanzania, many natural processes are intact but it is subjected to annual fire ‘management’ which might decrease populations of vectors such as ticks, thereby affecting transmission of tick-borne parasite infections in hyenas. We thus compared differences in the relative frequency of pathogen infections between brown and spotted hyenas within one geographic region (Namibia) and in one hyena species (spotted hyena) between two regions, which differed in the type and scale of human activities.

The brown hyena is listed as “Near Threatened” by the International Union for Conservation of Nature (IUCN), and its range is limited to arid and semi-arid regions in southern Africa [17]. Brown hyenas prey on small mammals including livestock, and also scavenge on carcasses of livestock and large wild herbivores, including those killed by other carnivores [18, 19]. We screened samples from brown hyenas that inhabited livestock grazing areas, where they were likely to encounter vectors of pathogens that infect livestock, companion animals associated with farms, and wildlife species that inhabit livestock farmland [20, 21]. Brown hyenas form small clans comprising individual females with their offspring, sometimes belonging to different age classes [22]. All females within a clan reproduce and mate with nomadic or immigrant males [18]. Clans hold large territories between 235 and 480 km^2^ [22].

The spotted hyena is listed as of “Least Concern” by IUCN. The spotted hyena samples in our study came from two separate populations in large protected areas, Etosha National Park (NP) in Namibia, which covers 22,270 km^2^, and Serengeti NP in Tanzania, which covers 14,763 km^2^. In Etosha NP, clan sizes are an order of magnitude smaller and clan territories an order of magnitude larger [23] than those in the Serengeti NP [24, 25]. In both populations, clans keep their cubs in communal dens. Communal den areas are social centres for clans, hence important locations for the transmission of pathogens [26–28]. The number of cubs occupying a den can increase the prevalence of vector-borne pathogens, as shown for the helminth *Dipylidium* sp. [29]. This suggests that individuals in the large ‘Serengeti’ clans should be more prone to pathogen infection than those in small ‘Etosha’ clans. Spotted hyenas with larger lifetime ranges are more likely to encounter pathogens [30] than those with more limited ranges. As a result of the migratory movement of herbivores in both Etosha and Serengeti NPs, adults in both populations forage over extensive areas [31, 32]. Etosha NP receives annual rainfall of 350 mm, far lower than the Serengeti NP with 1200 mm in the north-west and 500 mm in the south-east [32, 33], which suggests that parasite vectors with poor survival in arid conditions with high daytime temperatures may be less abundant in Etosha during the dry season. Etosha NP (unlike the Serengeti NP) has artificial waterholes, which attract water-dependent species [34]. This is likely to increase the abundance of vectors such as ticks in these locations in the vicinity of waterholes, and also vectors with life stages that require an aquatic environment, such as mosquitoes, which could breed throughout the dry season. In the Serengeti NP, large areas are burnt soon after the end of the long rains, which is not the case in Etosha NP. ‘Early burning’ should decrease tick abundance in burnt areas [35] and thereby reduce the transmission of tick-borne parasites. Hence infection with tick-borne parasites should be lower in spotted hyenas in the Serengeti NP than in Etosha NP. Brown hyenas in farmland that is not burnt and contains livestock, companion animals, wild herbivores and artificial water supplies for livestock may be expected to encounter the largest diversity of vectors and have the highest diversity of parasites.

This study focused on several vector-borne bacteria (Anaplasmataceae, *Rickettsia* spp.), Apicomplexa (*Hepatozoon* spp., *Babesia* spp.) and filarioid nematodes (Onchocercidae). Ticks (Ixodida) are the vectors for all *Babesia* species and all *Hepatozoon* species of carnivores and for most Anaplasmataceae and *Rickettsia* spp. [36]. In contrast, the vector spectrum of Onchocercidae is very broad, and different parasite species use different vectors including mosquitoes (Culicidae), black flies (Simuliidae), biting midges (Ceratopogonidae), horse flies (Tabanidae), fleas (Siphonaptera), louse flies (Hippoboscidae), lice (Phthiraptera), ticks and other mites such as Macronyssidae [36–40]. These parasites have stages that circulate in peripheral blood and can be readily detected by polymerase chain reaction (PCR). Currently, available information on vector-borne pathogens is sparse for both species of hyenas, particularly regarding bacterial pathogens. As far as we know, infection with Anaplasmataceae has not been previously reported in either brown or spotted hyenas. There are two studies where samples from brown and spotted hyenas from Zambia, Namibia and South Africa tested negative for *Ehrlichia* [41, 42]. For *Rickettsia* spp. there is a single serological study which stated that one of three investigated spotted hyenas was positive for antibodies against *Rickettsia akari* in a complement fixation assay at a dilution of 1:10 but not at a dilution of 1:40 [43]. No antibodies were detected against *Rickettsia conori*, *Rickettsia mooseri* or *Rickettsia burneti* [43].

Spotted hyenas are often infected with *Hepatozoon* sp. [44]. In the Serengeti NP, these parasites were highly similar or identical to *Hepatozoon felis* [45]. In Zambia, spotted hyenas were positive for both *Hepatozoon canis* and *H. felis* [41]. Williams et al. [41] also identified a *Babesia* sp. in spotted hyenas very similar to *Babesia lengau* which had been previously described in cheetahs (*Acinonyx jubatus*) in Namibia [46]. This finding was confirmed in a recent study from Namibia and South Africa for brown and spotted hyenas [42]. *Babesia lengau* or very similar *Babesia* spp. have been associated with severe disease in domestic cats and a sheep [47], which suggests that all the apicomplexan blood parasites described for brown and spotted hyenas might be a potential health risk to wild as well as domestic animals.

In the Turkana district in northern Kenya, two spotted hyenas were reported to be positive for *Acanthocheilonema dracunculoides* (syn. *Dipetalonema dracunculoides*) [48]. This parasite was also found in two domestic dogs in the same area and in domestic dogs in Namibia [49]. In both studies the parasites were identified either by morphometry of microfilaria [48] or by acid phosphatase staining [49], and therefore the exact species identification should be considered as doubtful without morphological data on adult parasites or DNA sequence data. Third-stage infective larvae with a morphology in accordance with *A. dracunculoides* were found in *Hippobosca longipennis* louse flies in Kenya [38].

In summary, at least four vector-borne parasites previously identified in brown hyenas (*H. canis*, *H. felis*, *B. lengau* and *A. dracunculoides*) are also potential pathogens in domestic animals. The present study thus aimed to identify vector-borne pathogens in the groups Anaplasmataceae, *Rickettsia* sp., *Hepatozoon* sp., Piroplasmida and Onchocercidae in brown hyenas from Namibia and spotted hyenas from Namibia and Tanzania.

## Methods

### Study area, sample collection and DNA extraction

In Namibia, 16 whole blood samples and one liver sample were collected from brown hyenas from farmland in central and north-central Namibia (*n* = 15) and farmland adjacent to the Skeleton Coast NP in north-west Namibia (*n* = 2). From spotted hyenas, 14 whole blood samples and five buffy coat samples were collected from the Etosha NP (*n* = 18) and the farmland in north-central Namibia (*n* = 1, Additional file 1: Table S1). The hyenas were captured, anaesthetised and sampled as previously described [50–52], except for one brown hyena shot by a farmer because he judged the animal to be a threat to his livestock. Samples from brown and spotted hyenas were stored in liquid nitrogen containers or at −20° until transport to Germany. In Germany, samples were stored at −80 °C before DNA extraction using the GEN-IAL^®^ First-DNA all-tissue kit. Blood samples from spotted hyenas (*n* = 25) were collected in the Serengeti NP in northern Tanzania from carcasses of recent vehicle accidents and predation by lions (*Panthera leo*), or from animals anaesthetised for the removal of wire snares [31]. These samples were stored at −20 °C until transport to Germany. DNA was extracted using the Maxwell^®^ 16 LEV Blood DNA Kit and the Maxwell^®^ 16 instrument (Promega). All DNA samples were stored at −20 °C or −80 °C until used for this study.

### PCR amplifications and sequencing

PCR was performed using either Phusion Hot Start II High-Fidelity DNA Polymerase or Maxima Hot Start Taq DNA Polymerase (both Thermo Scientific). All reactions except for the one targeting *Hepatozoon* spp. were conducted with Phusion enzyme in a 20 µL reaction volume consisting of 0.2 mM of deoxynucleoside triphosphates (dNTPs), 0.25 µM of each primer, 0.02 U/µL of Phusion Hot Start II High-Fidelity DNA Polymerase and 2 µL of template DNA in 1× Phusion HF Buffer. The *Hepatozoon* PCR was performed in 25 µL reaction volume consisting of 0.2 mM dNTPs, 0.3 µM of each primer, 0.04 U/µL Maxima Hot Start Taq DNA Polymerase, 2.5 mM MgCl_2_ and 2 µL of template DNA in 1× Maxima Hot Start PCR Buffer. PCR-specific denaturation and annealing temperatures and times used for the different PCR steps are provided in Additional file 2: Table S2. The number of PCR cycles was 40 except for the PCR to detect Anaplasmataceae, for which 50 cycles were conducted. For negative controls, nuclease-free water was used instead of template DNA in all PCR runs. As positive controls, plasmid DNA containing the respective amplicon was used. Primers used for pathogen-specific PCRs were derived from previous publications [53–60] and are provided in Additional file 2: Table S2.

A PCR specific for *B. lengau*-like parasites was developed. Primers were designed to match *B. lengau*, *B. lengau*-like parasites from hyenas and closely related parasites such as *B. conradae* and *Babesia duncani*. PCR conditions were optimised using samples known to be positive for *B. lengau*-like parasites and negative for *Hepatozoon* spp. or the classical *Babesia* species *Babesia vogeli* and *Babesia canis.* Evaluation of specificity was performed with samples negative for *B. lengau*-like piroplasms but positive for *B. canis*, *B. vogeli* or *H. canis*. The optimised PCR contained 0.25 µM of each primer, 0.2 mM dNTPs, 0.02 U/µl Phusion Hot Start II High-Fidelity DNA Polymerase and 2 µl template DNA in 20 µl Phusion HF buffer. Primer sequences are also provided in Additional file 2: Table S2. After an initial denaturation at 98 °C for 30 s, 40 cycles of denaturation at 98 °C for 20 s, annealing at 65 °C for 30 s and elongation at 72 °C for 30 s were conducted. Finally, reactions were incubated at 72 °C for 5 min. As positive control, DNA from a *B. lengau*-like blood sample was chosen.

For further characterisation of parasites, some amplification products from samples that showed positive results were purified with the DNA Clean & Concentrator^®^-5 Kit (Zymo Research Corporation, Irvine, USA) according to the manufacturer's instructions. If available, at least five samples were chosen for each of the three groups of hyenas, and very weak bands were avoided whenever possible. Then, purified PCR products were cloned into the StrataClone blunt-end PCR cloning vector ‘pSC-B-amp/kan’ supplied in the StrataClone Blunt PCR cloning kit (Agilent Technologies, CA, USA) or the TOPO TA Cloning Kit for Sequencing (Thermo Scientific), and recombinant plasmid vectors were transformed into Solopack1 (Agilent Technologies, CA, USA) or One Shot TOP10 (Thermo Scientific) competent cells according to the manufacturer's instructions. Plasmid DNA was isolated using the EasyPrep1Pro Plasmid Mini Prep Kit (Biozym, Oldendorf, Germany), and clones with inserts were sequenced by LGC Genomics (Berlin, Germany).

### Sequence analyses

Initially, sequences were compared to the NCBI GenBank™ database using BLASTn (https://blast.ncbi.nlm.nih.gov/Blast.cgi) reporting the percentage of identical nucleotides in the alignment [61]. If sequences in GenBank™ were more than 98% identical to our sequences, we considered this pathogen to belong to the species referred to in GenBank. If there was not a closely matched sequence available, the sequence was considered to belong to a species for which the respective target sequence had not been deposited in GenBank. In these cases, phylogenetic analyses were conducted to identify the closest known relatives of the pathogen.

### Phylogenetic analyses

All rRNA sequences were aligned using MAFFT with the Q-INS-i option [62, 63]. For 18S rRNA regions, the “Try to align gappy regions anyway” option was used (command line: mafft-qinsi-maxiterate 2-reorder input), while for internal transcribed spacer (ITS) sequences the “Leave gappy regions'' option was chosen (command line: mafft-qinsi-maxiterate 2-reorder-leavegappyregion input). Identification of best substitution models and calculation of best phylogenetic tree were both performed using ModelFinder [64] and IQ-TREE [65] on the IQ-TREE web server [66]. ModelFinder was set to autoselect the optimal substitution model and to also consider FreeRate heterogeneity models. Ultrafast bootstrapping [67] and Shimodaira–Hasegawa approximate likelihood ratio tests (SH-aLRTs) [68] were both performed with 1000 replicates. The final command line was: path_to_iqtree-s iqtree_Alignment.phy-st DNA -m TESTNEW-bb 1000-alrt 1000. Trees were visualised using FigTree version 1.4.4.

### Statistical analyses

For relative frequencies of pathogens, 95% confidence intervals (CIs) were calculated as Wilson score values using the binom.wilson function of the R package epitools 0.5-10 in R version 3.5.3. Differences in relative frequencies were judged to be significant with the help of the mid-P exact test conducted with the tab2by2.test function implemented in epitools. For all comparisons between the three groups of hyenas, *p* values were adjusted using the p.adjust method in R applying the “Holm” correction method. The number of pathogens simultaneously infecting an individual (co-infections) was compared between brown hyenas from Namibia (BHNA), spotted hyenas from Namibia (SHNA) and spotted hyenas from Tanzania (SHTZ) using a Kruskal–Wallis test followed by Dunn’s post hoc test in GraphPad Prism 5.03.

## Results

### Relative frequency and identification of Anaplasmataceae

A PCR previously shown to detect *Anaplasma* spp., *Ehrlichia* spp. and *Neoehrlichia* spp. was positive for 82.4%, 100.0% and 32.0% of BHNA, SHNA and SHTZ, respectively (Table 1). All relative infection frequencies were significantly different from each other (BHNA vs SHNA: *p* = 0.026, BHNA vs SHTZ: *p* = 0.003, SHNA vs SHTZ: *p* < 0.001). Sequence analyses for 22 out of 24 sequenced samples from all three hyena groups (7/7 BHNA, 10/10 SHNA, 5/7 SHTZ) revealed 98–100% identity with *Anaplasma platys*, *Anaplasma phagocytophilum* and *Anaplasma camelii* sequences (e.g., accession no. MN453481), which were identical in the amplified 16S rRNA gene fragments. For two SHTZ samples (I744 and C004), sequences were 98.1% identical with *Anaplasma bovis* (e.g., accession no. MH255940).

**Table 1:** Prevalence of infection with different vector-borne pathogens in brown and spotted hyenas from Namibia and spotted hyenas from Tanzania

Pathogen	Brown hyenas Namibia (BHNA)				Spotted hyenas Namibia (SHNA)				Spotted hyenas Tanzania (SHTZ)				Significance of comparisons (*p*^a^)		
	*n*	*N*	Freq. (%)	95% CI (%)	*n*	*N*	Freq. (%)	95% CI (%)	*n*	*N*	Freq. (%)	95% CI (%)	BHNA vs SHNA	BHNA vs SHTZ	SHNA vs SHTZ
Onchocercidae	7	17	47.1	26.2–69.0	9	19	47.4	27.3–68.3	9	25	36.0	20.2–55.5	1.000	1.000	1.000
Piroplasms	13	17	76.5	52.7–90.4	11	19	57.9	36.3–76.9	7	25	28.0	14.3–47.6	0.266	0.008	0.111
*Babesia lengau*	12	17	70.6	46.9–86.7	16	18	88.9	67.2–96.9	8	25	32.0	17.2–51.6	0.213	0.036	0.001
*Hepatozoon* spp.	11	17	64.7	41.3–82.7	7	19	36.8	19.1–59.0	11	25	44.0	26.7–62.9	0.337	0.416	0.650
Anaplasmataceae	14	17	82.4	59.0–93.8	19	19	100.0	83.2–100.0	8	25	32.0	17.2–51.6	0.026	0.003	< 0.001
*Rickettsia* spp.	1	17	5.9	1.0–27.0	3	19	15.8	5.5–37.6	0	25	0.0	0.0–13.3	0.810	0.810	0.219

*n*: number of positive samples; *N*: number of analysed samples; Freq.: frequency; 95% CI: 95% confidence interval

^a^mid-P exact test results after correction using the Holm method

### Relative frequency and identification of *Rickettsia* spp.

A *glt*A PCR was positive for one BHNA (5.9%), three SHNA (15.8%) and none of the SHTZ samples (Table 1), with no significant differences between the groups. Sequences of all four positive samples were 100% identical to *Rickettsia raoultii* (e.g., GenBank accession no. MN550896). Since the number of positive samples was low and the identified species was the same as the one used as positive control, the positive control plasmids obtained by cloning from German *Dermacentor reticulatus* tick samples [69] were re-sequenced. All sequences from Namibian samples consistently differed by a single nucleotide from the positive control, excluding the possibility of a false-positive diagnosis.

### Relative frequency and phylogenetic position of identified *Hepatozoon* spp.

A PCR targeting the 18S rRNA of *Hepatozoon* spp. was conducted for all samples. Frequencies of infection with *Hepatozoon* spp. were 64.7%, 36.8% and 44.0% for BHNA, SHNA and SHTZ, respectively (Table 1). Relative frequencies did not differ significantly between the three groups (mid-P exact test).

**Fig. 1:**
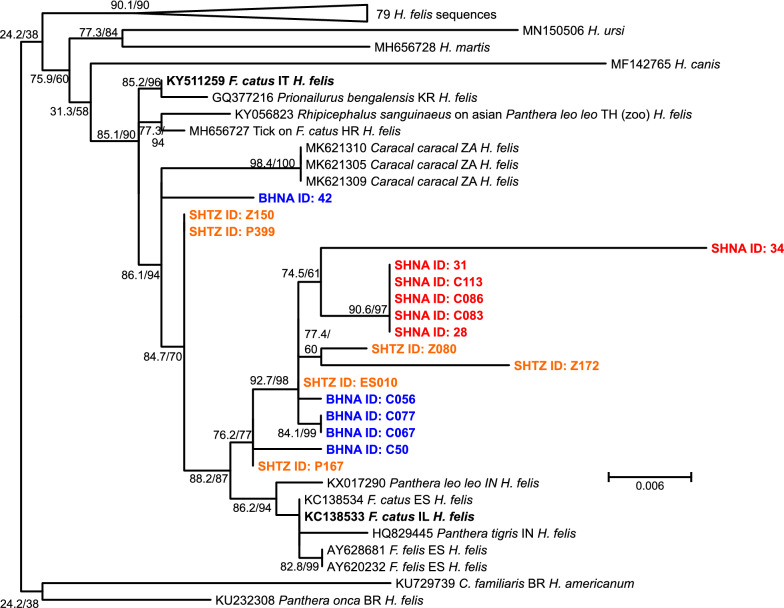
Maximum-likelihood phylogenetic tree calculated from Hepatozoon (H.) spp. 18S ribosomal rRNA sequences. Spotted hyenas from Namibia (SHNA) and Tanzania (SHTZ) are highlighted in red and orange, respectively, while brown hyenas from Namibia (BHNA) are coloured in blue. The host species name is abbreviated for dogs (Canis lupus familiaris = C. familiaris) and domestic cats (Felis silvestris catus = F. catus) while the host species are provided with the complete name: leopard cat (Prionailurus bengalensis), brown dog tick (Rhipicaphalus sanguineus), caracal (Caracal caracal), lion (Panthera leo leo), tiger (Panthera tigris), jaguar (Panthera onca). Node support values represent the results of the Shimodaira–Hasegawa approximate likelihood ratio tests before and of ultrafast bootstrapping behind the slash. The scale bar represents 0.04 substitutions per site. The country from which the samples originate are indicated using the ISO 3266-2 two letter code: BR: Brazil; ES: Spain; IL: Israel; IN: India; IT: Italy; TH: Thailand. The ID values for the hyenas from this study correspond to the IDs of individual animals listed in Additional file 1: Table S1

BLASTn analyses identified as best hits either the sequence KY511259 (97.1% to 99.5% identity) or KC138533 (98.4% to 99.4% identity), both annotated in GenBank as *H. felis* derived from domestic cats. Since the identity of some sequences to the best hit from *H. felis* was below 99%, a phylogenetic analysis was conducted. This analysis did not confirm that the sequences identified from brown or spotted hyenas belonged to *H. felis*. Although the phylogram in Fig. 1 is characterised by generally low node support values, none of the 17 sequences from hyenas (five BHNA, six SHNA, six SHTZ) was included in the large cluster of 79 sequences assigned to the taxon *H. felis* (designated as *H. felis* sensu stricto for the discussion). The GenBank entries KY511259 and KC138533 were also not included in this cluster.

### Relative frequency and phylogenetic position of identified Piroplasmida

In a first step, a published pan-piroplasm PCR [55, 58] was used to analyse all hyena samples. In this PCR, 76.5%, 57.9% and 28.0% of the BHNA, SHNA and SHTZ samples were positive, respectively (Table 1). The relative frequency of infection in SHTZ was significantly lower than in BHNA (*p* = 0.008, mid-P exact test); there was no significant difference between the other paired comparisons (Table 1).

Sequence analyses of PCR products revealed that one out of 15 samples represented *Sarcocystis* spp. With the exception of a single *B. vogeli*-like sequence (96.9% identity with MK830995), all piroplasm sequences showed 94.4% to 97.5% identity with *B. lengau* or *B. lengau*-like parasites (*n* = 13).

The new PCR suitable for detecting the *B. lengau*/*B. conradae* group was applied to all samples except for four SHNA samples from which no DNA was left (this PCR was conducted as the last one). Since sequence data for three of these samples for the pan-piroplasm PCR confirmed the presence of *B. lengau*-like parasites, these samples were considered to be positive for this parasite, whereas the only positive sample in the pan-piroplasm PCR which was not sequenced was considered to have an unidentified status. Relative frequencies of *B. lengau*-like parasites in BHNA, SHNA and SHTZ were 70.6%, 88.9% and 32.0%, respectively (Table 1). The relative frequency of infection in SHTZ was significantly lower than that in SHNA (*p* = 0.001, mid-P exact test) and BHNA (*p* = 0.036). There was no significant difference between the hyena species in Namibia (Table 1).

**Fig. 2:**
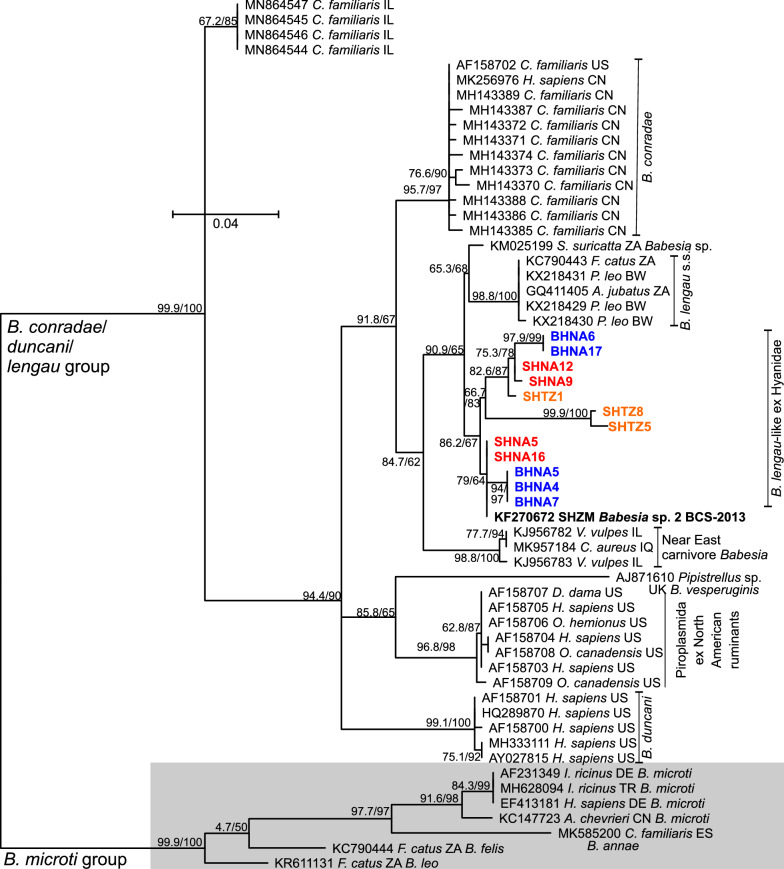
Maximum-likelihood phylogenetic tree calculated from *Babesia* (*B.*) spp. from the *Babesia conradae/duncani/lengau* group using partial 18S ribosomal rRNA sequences. The outgroup (light grey square) consists of several species from the *Babesia microti* group. Spotted hyenas from Namibia (SHNA) and Tanzania (SHTZ) are highlighted in red and orange, respectively, while brown hyenas from Namibia (BHNA) are coloured in blue. The host species name is abbreviated for dogs (*Canis lupus familiaris* = *C. familiaris*) and domestic cats (*Felis silvestris catus* = *F. catus*) while the host species are provided with the genus name abbreviated: meerkat (*Suricata suricatta*), lion (*Panthera leo*), cheetah (*Acinonyx jubatus*), red fox (*Vulpes vulpes*), golden jackal (*Canis aureus*), a bat (*Pipistrellus* sp.), fallow deer (*Dama dama*), humans (*Homo sapiens*), mule deer (*Odocoileus hemionus*), bighorn sheep (*Ovis canadensis*), castor bean tick (*Ixodes ricinus*), Chevrier's field mouse (*Apodemus chevrieri*). Node support values represent the results of the Shimodaira–Hasegawa approximate likelihood ratio tests before and of ultrafast bootstrapping behind the slash. The scale bar represents 0.04 substitutions per site. The country from which the samples originate are indicated using the ISO 3266-2 two letter code: BW: Botswana; CN: China; DE: Germany; ES: Spain; IL: Israel; IQ: Iraq; TR: Turkey; UK: United Kingdom; US: United States of America; ZA: South Africa. The ID values for the hyenas from this study correspond to the IDs of individual animals listed in Additional file 1: Table S1

Since the sequences obtained from the pan-piroplasm PCR are quite short, no analysis over all piroplasms was conducted. Instead, two separate analyses were conducted for the *B. lengau*- and the *B. vogeli*-like sequences. For *B. lengau*-like piroplasms, similar sequences in GenBank were selected on the basis of high identity in BLASTn. For the phylogram, only one selected sequence of *Babesia vesperuginis* (*B. conradae*/*duncani*/*lengau* group) was included. For the outgroup, sequences of *Babesia leo*, *Babesia felis*, *Babesia annae* and *Babesia microti* (all belonging to the *Babesia microti*-like group) were used. The phylogram in Fig. 2 presents the results of the maximum-likelihood phylogenetic analysis and reveals that all sequences from hyenas from the present study as well as from a spotted hyena from Zambia form a single clade (*B. lengau*-like ex Hyaenidae). This clade shows only moderate statistical support and appears to be quite divergent (Fig. 2). The hyena parasites were closely related to a homogeneous and strongly supported group of *B. lengau* s.s. from wild and domestic felines from southern Africa and to a single sequence reported from a meerkat (*Suricata suricatta*) from Zambia. In comparison to *B. lengau*-like ex Hyaenidae, all other groups of *B. conradae*/*duncani*/*lengau* showed much lower intra-group variability, suggesting that *B. lengau*-like ex Hyaenidae might consist of more than one species (Fig. 2).

**Fig. 3:**
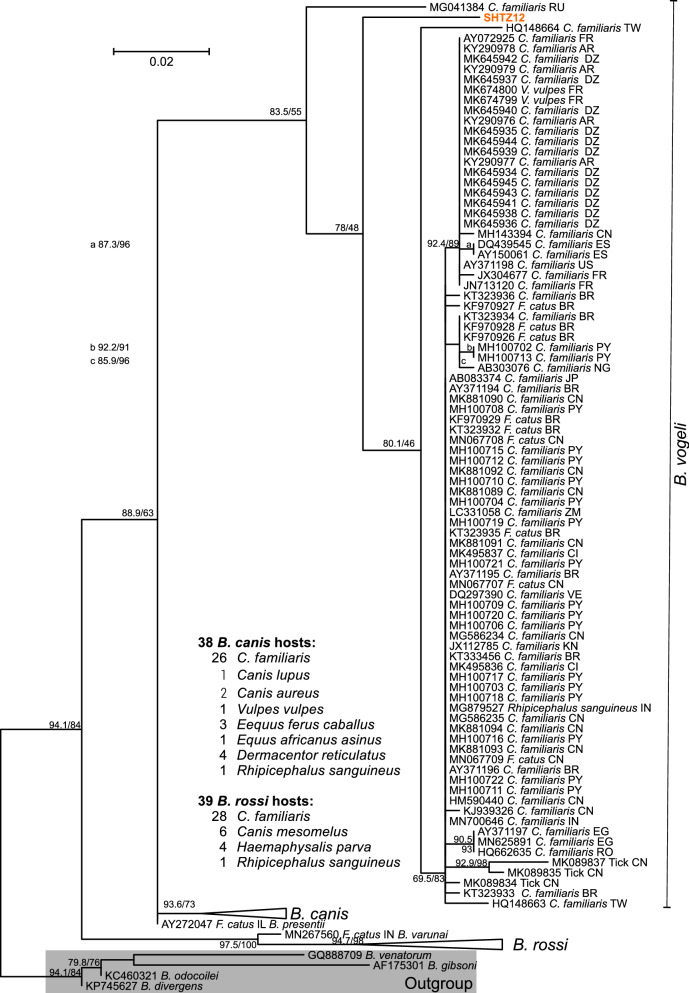
Maximum-likelihood phylogenetic tree calculated from *Babesia* (*B.*) spp. from the *Babesia canis* group using partial 18S ribosomal rRNA sequences. The outgroup (light grey square) consists of several species from the *Babesia* sensu stricto group. Spotted hyenas from Namibia (SHNA) and Tanzania (SHTZ) are highlighted in red and orange, respectively, while brown hyenas from Namibia (BHNA) are coloured in blue. The host species name is abbreviated for dogs (*Canis lupus familiaris* = *C. familiaris*) and domestic cats (*Felis silvestris catus* = *F. catus*) while the brown dog tick (*Rhipicephalus sanguineus*) is provided with the complete name. Node support values represent the results of the Shimodaira–Hasegawa approximate likelihood ratio tests before and of ultrafast bootstrapping behind the slash. The scale bar represents 0.02 substitutions per site. The country from which the samples originate are indicated using the ISO 3266-2 two letter code: AR: Argentina; BR: Brazil; CI: Cote d'Ivoire; CN: China; DZ: Algeria; EG: Egypt; ES: Spain; FR: France; IN: India; KN: Saint Kitts and Nevis; NG: Nigeria; JP: Japan; PY: Paraguay; RO: Romania; RU: Russia, TW: Taiwan; US: United States of America; VE: Venezuela; ZM: Zambia. The ID values for the hyenas from this study correspond to the IDs of individual animals listed in Additional file 1: Table S1

To identify the phylogenetic position of the single *B. vogeli*-like sequence identified in a SHTZ, all *B. vogeli*, *B. canis*, *B. rossi*, *Babesia presentii* and *Babesia varunai* sequences with a query coverage of at least 98% were included. These five species were previously considered to represent subspecies of *B. canis*, but at least the previous subspecies *B. canis canis*, *B. canis vogeli* and *B. canis rossi* are now considered to be independent species (Solano-Gallego and Baneth, 2011). As an outgroup, sequences of *Babesia gibsoni*, *Babesia divergens*, *Babesia odocoilei*, and *Babesia venatorum* (all *Babesia* s.s.) were chosen. The vast majority of the *B. vogeli* sequences form a single cluster with very little variability (Fig. 3). Two sequences annotated as *B. vogeli* from domestic dogs and the sequence from the SHTZ were located closer to the origin of the tree than the large cluster. The three sequences did not cluster with each other. They were in principle in a similar position as (i) *B. presentii* in comparison to *B. canis* and (ii) *B. varunai* in comparison to *B. rossi* and might represent either additional species or subspecies of *B. vogeli* (Fig. 3).

### An undescribed species of the genus *Sarcocystis*?

The samples were not systematically screened for the presence of *Sarcocystis*, since this parasite is only transiently present in peripheral blood. Because of cross-reactivity of the pan-piroplasm PCR, a single sequenced clone obtained from a SHNA represented a *Sarcocystis* sequence that was most similar (95.9%) to *Sarcocystis* sp. isolate S.MonN1Oc14 amplified from muscle tissue of a white-tailed mongoose (*Ichneumia albicauda*) from South Africa. The second best matching species was *Sarcocystis nesbitti*, a zoonotic species from Southeast Asia assumed to use pythons as the definitive and primates as intermediate hosts. Since the 18S rRNA target is highly conserved, and pairwise sequence identity between 15 sequences annotated as *S. nesbitti* was always > 97.5%, it is likely that the new sequence is a species not previously represented in GenBank.

**Fig. 4:**
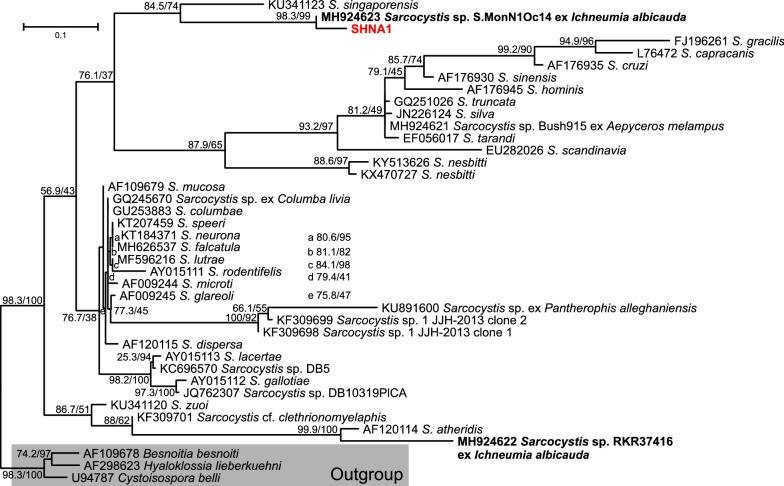
Maximum-likelihood phylogenetic tree calculated from *Sarcocystis* (*S.*) spp. using partial 18S ribosomal rRNA sequences. The outgroup (light grey square) consists of several species of Coccidia. Spotted hyenas from Namibia (SHNA) and Tanzania (SHTZ) are highlighted in red and orange, respectively, while brown hyenas from Namibia (BHNA) are coloured in blue. The host species name is given only for species infecting carnivores from southern Africa, i.e. white-tailed mongoose (*Ichneumia albicauda*), printed in bold, or if the name was part of the provisional *Sarcocystis* species name. Node support values represent the results of the Shimodaira–Hasegawa approximate likelihood ratio tests before and of ultrafast bootstrapping behind the slash. The scale bar represents 0.1 substitutions per site. The ID values for the hyenas from this study correspond to the IDs of individual animals listed in Additional file 1: Table S1

Phylogenetic analyses confirmed that the parasite, in the following designated as *Sarcocystis* sp. ex *Crocuta crocuta*, is in a sister position to *Sarcocystis* sp. isolate S.MonN1Oc14 from *I. albicauda* (Fig. 4). The second closest relative was not *S. nesbitti* but *Sarcocystis singaporensis*, another species which uses pythons as definitive and rodents as intermediate hosts. Grouping of *S. singaporensis* with the two species using SHNA and white-tailed mongoose from southern Africa as intermediate hosts had only moderate statistical support (Fig. 4). The only other *Sarcocystis* species described using carnivores from South Africa as intermediate hosts, also from a white-tailed mongoose, was not closely related to *Sarcocystis* sp. ex *Crocuta crocuta* (Fig. 4).

### Relative frequency and phylogenetic position of the identified Onchocercidae

The ITS-2 PCR for filarioid nematodes revealed high relative frequencies of 47.1%, 47.4% and 36.0% in BHNA, SHNA and SHTZ, respectively (Table 1). None of the comparisons between relative frequencies in the three groups was significant. These results suggest that transmission of filarioids is similar across study sites in Namibia and Tanzania.

**Fig. 5:**
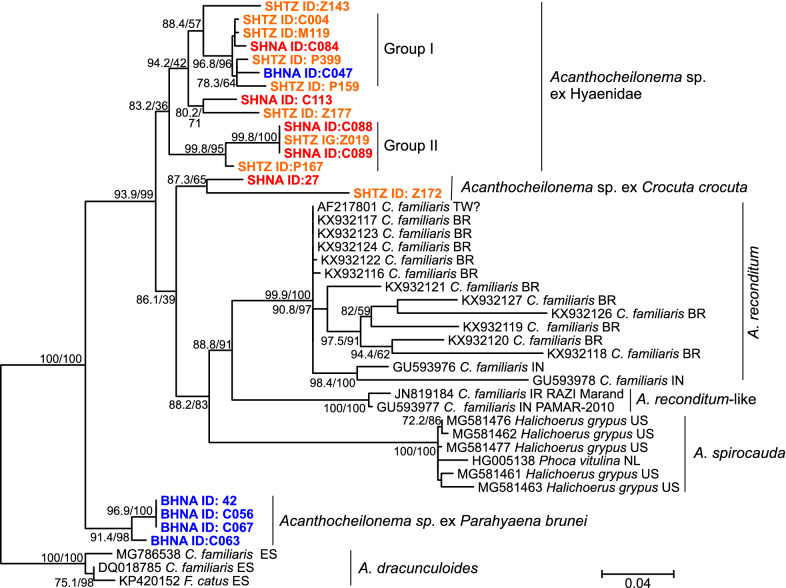
Maximum-likelihood phylogenetic tree calculated from Acanthocheilonema spp. ITS-2 ribosomal rRNA sequences. Spotted hyenas from Namibia (SHNA) and Tanzania (SHTZ) are highlighted in red and orange, respectively, while brown hyenas from Namibia (BHNA) are coloured in blue. The host species name is abbreviated for dogs (Canis lupus familiaris = C. familiaris) and domestic cats (Felis silvestris catus = F. catus) while the other host species are provided with the complete name: grey seal Halichoerus grypus and harbour seal Phoca vitulina. The sequences labelled as A. reconditium-like are assigned to the species Acanthocheilonema sp. RAZI Marand and Acanthocheiloneme sp. PAMAR-2010 in GenBank. Node support values represent the results of the Shimodaira–Hasegawa approximate likelihood ratio tests before and of ultrafast bootstrapping behind the slash. The scale bar represents 0.04 substitutions per site. The country from which the samples originate are indicated using the ISO 3266-2 two letter code: BZ, Brazil; ES, Spain; IN, India; IR, Iran; NL, Netherlands; TW, Taiwan; US, United States of America. The “?” indicates that the country of origin was not deposited in GenBank and the given value represents the origin of the authors. The ID values for the hyenas from this study correspond to the IDs of individual animals listed in Table S1.Maximum-likelihood phylogenetic tree calculated from Hepatozoon (H.) spp. 18S ribosomal rRNA sequences. Spotted hyenas from Namibia (SHNA) and Tanzania (SHTZ) are highlighted in red and orange, respectively, while brown hyenas from Namibia (BHNA) are coloured in blue. The host species name is abbreviated for dogs (Canis lupus familiaris = C. familiaris) and domestic cats (Felis silvestris catus = F. catus) while the host species are provided with the complete name: leopard cat (Prionailurus bengalensis), brown dog tick (Rhipicephalus sanguineus), caracal (Caracal caracal), lion (Panthera leo leo), tiger (Panthera tigris), jaguar (Panthera onca). Node support values represent the results of the Shimodaira–Hasegawa approximate likelihood ratio tests before and of ultrafast bootstrapping behind the slash. The scale bar represents 0.04 substitutions per site. The country from which the samples originate are indicated using the ISO 3166-2 two letter code. The ID values for the hyenas from this study correspond to the IDs of individual animals listed in Additional file 1: Table S1

Sequence data were obtained by choosing five, five and nine samples from BHNA, SHNA and SHTZ, respectively, followed by cloning and sequencing a single clone per sample. All revealed reasonable identity (82.9–86.2%) in BLASTn analyses to *Acanthocheilonema* spp. sequences in GenBank. These identities were much lower than would be expected for intraspecific comparisons. Identity between the new sequences was between 79.3 and 100%. Maximum-likelihood phylogenetic analysis was conducted by including all *Acanthocheilonema* sequences from GenBank as well as all 19 sequences obtained in the present study. We did not use any proper outgroups since there was virtually no sequence similarity of the ITS-2 region to members of the Onchocercidae outside the genus *Acanthocheilonema*. Therefore, the phylogram presented in Fig. 5 shows an unrooted tree. Because of its low intraspecific variability, *A. dracunculoides* was arbitrarily chosen as outgroup to improve visualisation. The inner nodes of the tree show only low to moderate statistical support; the phylogeny of the different groups has thus to be confirmed using additional markers. Nevertheless, the tree contains three strongly supported groups with low sequence variability and a considerable distance to other operational taxonomic units: *A. dracunculoides* from domestic dogs and domestic cats, the seal heartworm *Acanthocheilonema spirocauda* from earless seals (Phocidae) and four sequences exclusively detected in BHNA and representing four of five BHNA sequences (designated as *Acanthocheilonema* sp. ex *Parahyaena brunnea* in Fig. 5). These groups most likely represent valid species. The widely accepted species *Acanthocheilonema reconditum* was statistically highly supported, although intraspecific variability was very high, suggesting that sequences might have originated from more than one (cryptic) species. Most closely related to *A. reconditum* were two sequences reported from domestic dogs from northern India (Kashmir) and Iran labelled *A. reconditum*-like in Fig. 5. Two sequences from spotted hyenas from Namibia and Tanzania labelled *Acanthocheilonema* sp. ex *Crocuta crocuta* formed a separate group, but the length of the branches connecting these sequences was rather high and the statistical support for this group was only moderate. Most sequences obtained from spotted hyenas were located in the cluster labelled *Acanthocheilonema* sp.ex Hyaenidae (Fig. 5). It contains sequences obtained from spotted hyenas of both Namibian and Tanzanian origin and one sequence from a brown hyena in Namibia. The support for this cluster was low and variability was moderately high. There are two subgroups in this cluster containing six and four out of 13 *Acanthocheilonema* sp. ex Hyaenidae sequences, which show high statistical support (Group I and Group II in Fig. 5). It remains unclear whether this is of any taxonomic relevance.

### Co-infections with multiple vector-borne pathogens

**Fig. 6:**
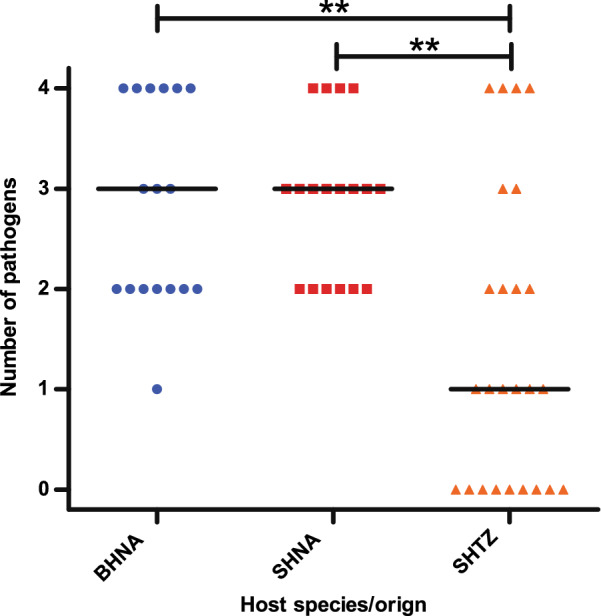
Number of pathogens detected in individual hyenas. Spotted hyenas from Namibia (SHNA) and Tanzania (SHTZ) are shown in red and orange, respectively, while brown hyenas from Namibia (BHNA) are coloured in blue. Horizontal lines indicate the medians. **p < 0.01 in a Kruskal–Wallis test followed by Dunn’s post hoc test

Most hyenas were infected by more than one vector-borne pathogen. The number of pathogens (*Acanthocheilonema* spp., *B. lengau*, *Hepatozoon* spp., *Anaplasma* spp., *Rickettsia* spp.) detected per individual and thus the extent of and species richness for co-infections is shown for BHNA, SHNA and SHTZ in Fig. 6. The median number of detected, simultaneously co-occurring pathogens was 3 (range 1–4) in BHNA, 3 (range 2–4) in SHNA and 1 (range 0–4) in SHTZ. The median for SHTZ was significantly lower than those for both hyena species from Namibia (both *p* < 0.01, Kruskal–Wallis test followed by Dunn’s post hoc test). In Namibia, brown (all from farmland) and spotted hyenas (all but one from Etosha NP) did not significantly differ in the number of co-infecting parasite types.

## Discussion

Ideally, to determine the prevalence of pathogens in wildlife species or populations, the samples used should be representative in terms of age, sex and other factors such as social status, but in reality this is often difficult to achieve. For example capturing wildlife to obtain blood samples may yield samples skewed in terms of age, sex or other factors. Also, invasive sampling methods may not be permitted or are limited concerning the number of individual that can be sampled, particularly in national parks. If so, samples collected from animals shortly after their natural death (e.g. those killed by vehicles or predators) can also provide useful insights into pathogen infection, but the results from relatively small numbers of animals should be interpreted with care. Our results come from two research projects that applied different sampling and DNA isolation methods. This may have resulted in different DNA yields and quality from the samples from these projects, which may affect the comparisons of frequencies of infection between spotted hyena populations in Namibia and Tanzania.

The frequency of Anaplasmataceae was very high in SHNA (100%) and BHNA (82.4%) but much lower in SHTZ (32.0%). In contrast, Burroughs et al. [42] did not detect any Anaplasmataceae in brown (*n* = 59) and spotted (*n* = 47) hyenas in Namibia and South Africa using a reverse-line blot approach. The reason for this striking difference is unclear. It might be a consequence of the different methods used, including the high number of PCR cycles used in the present study (*n* = 50), which is feasible with the Phusion DNA polymerase but not with most other polymerases. Generally, the relative frequency of detection of Anaplasmataceae in wild carnivores differed widely between various studies (range 0.0–57.7%) as reviewed by André [13]. Remarkably, the highest value of 57.7% was detected in South African black-backed jackals (*Canis mesomelas*) [70], whereas in African wild dogs also from South Africa none of 301 individuals was positive [71], with the reverse-line blot method being used in both studies. Strong differences in relative frequency of Anaplasmataceae were also observed in a recent study in caracals (*Caracal caracal*) from South Africa, with a prevalence of 88% in a periurban host population, and two rangeland populations showing 0% and 11% prevalence [72].

The relative frequencies of Anaplasmataceae in the Namibian BHNA and SHNA were considerably higher than in all previous studies except for the periurban caracals from South Africa, with frequencies between those of BHNA and SHNA, thus increasing the range of observed relative frequencies. The most likely explanation for the higher relative frequencies of infection in our Namibian study areas than in our study area in Tanzania are ecological differences between Southern Africa and East Africa, rather than a technical issue such as contamination, since positive samples were not clustered in particular PCR runs. In the absence of other studies from East Africa, we do not know whether the relatively low frequencies of infection in spotted hyenas from the Serengeti NP is representative of the prevalence in East African wild carnivores in general. In summary, our results and those from other studies indicate that the prevalence of Anaplasmataceae in carnivores in southern Africa can be high but also highly variable, and comparison of results produced by different methods may not be valid.

Our sequence data do not allow us to identify our *Anaplasma* sequences to the species level. Most sequences were equally similar to *A. platys, A. phagocytophilum* and *A. camelii*. Since the latter has so far only been found a few times and only in camelids and ruminants, it is unlikely to be this species. *Anaplasma platys and A. phagocytophilum* (which has a very broad host spectrum) are known to infect carnivores, but undescribed, closely related species cannot be excluded. *Anaplasma bovis* has been reported from racoons (*Procyon lotor*, family Procyonidae), coatis (*Nasua nasua*, family Procyonidae), racoon dogs (*Nyctereutes procyonoides*, family Canidae), crab-eating foxes (*Cerdocyon thous*, family Canidae) and a bush dog (*Speothos venaticus*, family Canidae) from the Carnivora suborder Caniformia and Tsushima leopard cats (*Prionailurus bengalensis euptilura*, family Felidae) and ocelots (*Leopardus pardalis*, family Felidae) from the suborder Feliformia [13]. Thus, *A. bovis* is frequently observed in carnivores, despite its name suggesting it is a parasite of cattle or more generally of ruminants, and finding *A. bovis*-like genotypes in spotted hyenas in Tanzania is therefore not unexpected.

The only *Rickettsia* species found in this study was *R. raoultii*. This species has been associated predominantly with *Dermacentor* spp. ticks in Eurasia, causing tick-borne lymphadenopathy/*Dermacentor*-borne necrosis erythema lymphadenopathy/scalp eschar neck lymphadenopathy (TIBOLA/DEBONEL/SENLAT) in humans [73, 74]. In *D. reticulatus*, prevalence exceeding 70% was observed in some locations [69], whereas direct detection of *R. raoultii* in non-human hosts has been rare. Liesner et al. [75] found seven out of 1023 (0.7%) domestic dog blood samples and none of 195 red fox (*Vulpes vulpes*) spleen samples from Brandenburg (Germany) to be positive, despite *D. reticulatus* occurring in the area at high abundance and high prevalence of *R. raoultii* in the local tick population [58, 69, 76]. In wildlife, *R. raoultii* has been detected in one Mongolian gazelle (*Procapra gutturosa*) [77] and one marbled polecat (*Vormela peregusna*) [78] in China, two bank voles (*Myodes glareolus*) [79] and a common vole (*Microtus arvalis*) [80] in Germany and a wild boar (*Sus scrofa*) in Italy [81]. As far as we know, finding *R. raoultii* in brown and spotted hyenas is the first reported presence of this spotted fever *Rickettsia* species in sub-Saharan Africa. The vector tick responsible for transmission in Africa remains unclear. The only member of the genus *Dermacentor* known to occur in southern Africa is *Dermacentor rhinocerinus*. Adults of this parasite feed quite specifically on black (*Diceros bicornis*) and southern white (*Ceratotherium simum*) rhinoceros in southern Africa [82]; little is known about the hosts of the larvae and nymphs of *D. rhinocerinus*. In one study, larvae and nymphs were observed to feed on rodents [83]. Given this lack of information, transmission by immature *D. rhinocerinus* stages to hyenas cannot currently be excluded.

*Hepatozoon* spp. have been known to infect spotted hyenas in South Africa [44] and Tanzania [45]. East et al. [45] identified hepatozoonosis as an important cause of death in juvenile hyenas. In Zambia, both *H. felis*-like and *H. canis*-like sequences were amplified from spotted hyena samples [41]. As far as we know, the present study is the first to report and characterise *Hepatozoon* spp. from brown hyenas. Our phylogenetic analysis revealed that *Hepatozoon* spp. from both hyena species are not identical to the typical *Hepatozoon* genotype (*H. felis* s.s.) found in domestic cats. Most *Hepatozoon* species included in the analysis as outgroups, such as *H. canis*, *Hepatozoon ursi* or *Hepatozoon martis*, were all placed between the homogeneous *H. felis* s.s. group and the group containing sequences obtained from hyenas in this study. The latter are more diverse than the *H. felis* s.s. group and are located between sequences assigned to *H. felis* in GenBank^®^ obtained from domestic cats and wild felids. This suggests that assignment of the hyena parasites to the taxon *H. felis* should be considered with care. Analyses with full-length 18S rRNA and additional markers will be required to resolve the phylogenetic position with sufficient accuracy.

The primer pair we used to detect piroplasms detected *B. lengau* and *B. vogeli* in the samples. *Babesia lengau*, a close relative of *B. conradae* and *B. duncani*, is not a “classical” *Babesia* (*Babesia* sensu stricto) species like *B. canis* or *Babesia bovis. Babesia lengau* is very distantly related to the “classical” *Babesia* group and belongs to the so-called Western group of piroplasms [84], while *B. vogeli* belongs to the group of *Babesia* s.s. In addition to the expected *Babesia* species, *H. felis*-like and *Sarcocystis* spp. parasites were found. This primer pair is very widely used, since it is able to amplify all groups of piroplasms. However, it is also recognised to lack sufficient specificity for Piroplasmida due to some cross-reactivity with Coccidia [85] and *Hepatozoon* spp. Thus, methods such as sequencing or reverse-line blot must be applied to positive samples to confirm the presence of piroplasms and identify the species.

Furthermore, the primers for piroplasms did not exactly match the target sequences in *B. lengau*-like GenBank entries, suggesting not only limited specificity but also limited sensitivity for this particular group. To improve both specificity and sensitivity, a *B. lengau*/*B. conradae* group PCR was developed. Under the optimised PCR conditions, no cross-reactivity was observed. Sequence identity of the new primers targeting the *B. lengau*/*B. conradae* group to sequences from tissue-cyst-forming coccidia such as *Sarcocystis* or *Toxoplasma* was very low (maximum of 14 consecutive identical bases for the 25 bp specific forward primer and no perfect identities at the 3′ end of the primer), and an amplification of these parasites was considered to be unlikely at an annealing temperature of 95 °C. Sequence analyses of initially amplified samples did not show any hint that the optimised PCR would also pick up tissue-cyst-forming coccidians; all analysed sequences were of the *B. lengau* type. Using this PCR, the frequency of *B. lengau* was significantly lower in SHTZ than in both hyena species from Namibia, whereas a comparison between the two hyena species in Namibia revealed no significant difference. These results are not consistent with the expectation that anthropogenic factors on farmland in Namibia result in higher infection of brown hyenas than spotted hyenas in Etosha NP, but are consistent with the prediction of lower infection in spotted hyenas in the Serengeti NP than in both Namibian study areas. Furthermore, the comparatively small difference between hyenas inhabiting farmland and Etosha NP may indicate that relatively similar factors affect both areas (such as those associated with artificial sources of water) and perhaps a lower prevalence of infected ticks in farmland, where some farmers take measures to control ticks, other than large-scale burning of swards as in the Serengeti NP.

Burroughs et al. [42] sequenced nearly full-length 18S rRNA genes of *B. lengau*-like piroplasms from brown and spotted hyenas in Namibia and South Africa and identified four genotype groups. As their sequences have not been published and were not accessible, they could not be included in the analysis. *Babesia lengau*-like piroplasms were associated with severe clinical symptoms in two domestic cats in South Africa [86] and an outbreak of piroplasmosis in two domestic sheep flocks in Greece [47]. Clinical signs in both host species included severe haemolysis and anaemia. The sheep also showed haemoglobinuria, and one domestic cat showed considerable histopathological changes in the brain. Findings of *B. lengau*-like piroplasms in six spotted hyenas and a single lion (*Panthera leo*) from Zambia [41] suggest that these parasites have a large geographical distribution in southern Africa and a host range that might include many Feliformia. Very similar sequences were obtained from a golden jackal (*Canis aureus*) and four red foxes from Israel [87], labelled “Near East carnivore *Babesia*” in Fig. 2. With the exception of the Greek outbreak in sheep, all hosts belonged to the order Carnivora. Since species of the families Felidae, Hyaenidae, Herpestidae and Canidae were infected, investigating carnivore species of other families, particularly in Africa, might help to provide a more complete picture of the host range.

With the pan-piroplasm PCR, we identified a single *B. vogeli*-like sequence in a SHTZ. Although *B. vogeli* is widely considered to be a canine parasite, it was repeatedly demonstrated to occur in domestic cats from Brazil, China, Portugal, Qatar and Thailand [13, 88–92]. In Spain, the parasite was also detected in stone martens (*Martes foina*) using real-time PCR and applying a *B. vogeli*-specific probe [93]. Such data suggest that *B. vogeli* and closely related genotypes have a wide host range and can also be expected to be present in other wild carnivore species.

With the pan-piroplasm PCR we also identified a *Sarcocystis* spp. sequence in one SHTZ. Carnivores are often definitive hosts of *Sarcocystis* spp. but a few species also use carnivores as intermediate hosts, such as *Sarcocystis caninum* and *Sarcocystis svanai* in domestic dogs and *Sarcocystis arctica* in arctic foxes (*Vulpes lagopus*) and red foxes [94–98]. Since the life cycle includes stages that infect mononuclear cells, finding *Sarcocystis* spp. in blood samples during acute infections is not surprising and has been previously reported [75]. As a method to estimate prevalence, PCR analysis of blood samples is unsuitable, since the prevalence in muscle tissue of chronically infected animals will be far higher than the prevalence in blood. The phylogenetic analysis placed this *Sarcocystis* sp. ex *Crocuta crocuta* in a sister position to a *Sarcocystis* from a white-tailed mongoose from South Africa. This suggests that at least closely related *Sarcocystis* species might use several Carnivora in Southern Africa as intermediate host. To identify the parasite species and their intermediate and definitive host specificity, systematic screening of muscle samples would be required.

The genetic variability of the *Acanthocheilonema* sequences obtained from hyenas was very high. These apparently represent several previously unrecognised species. In order to resolve them taxonomically, combined morphological and molecular analyses of adult worms will be required. For *D. repens*, multiple cryptic species were identified which come from different geographic locations and may also have different host ranges, suggesting that species diversity of filarioid parasites is considerably underestimated [99–101]. For strongyle nematodes, the combination of morphological and molecular data revealed synonyms [102, 103] as well as cryptic species [104, 105].

This also suggests that previous reports of *A. dracunculoides* in spotted hyenas and domestic dogs in Kenya [48] should be treated with caution. It might be that the microfilaria of two different species infecting hyenas and domestic dogs were morphologically undistinguishable from *A. dracunculoides* and that only the domestic dogs were infected with *A. dracunculoides.* Alternatively, spotted hyenas and domestic dogs in Kenya might be infected by an *Acanthocheilonema* species similar to *Acanthocheilonema* sp. ex Hyaenidae. To discriminate between these possibilities, systematic blood sampling and PCR/sequencing analyses of blood from hyena and domestic carnivores living in overlapping habitats would be required. This should also include efforts to identify potential vector species. As mentioned above, the spectrum of vectors for filarioids is broad. In the genus *Acanthocheilonema* this variation in vectors is particularly high. While the rodent parasite *Acanthocheilonema viteae* uses the soft tick *Ornithodorus tartakovskyi* [106], *A. dracunculoides* infecting domestic dogs is transmitted by the brown dog tick *Rhipicephalus sanguineus* [107, 108] and the louse fly *H. longipennis* [38]. The other *Acanthocheilonema* species commonly found in domestic dogs, *A. reconditum*, uses various fleas (including *Ctenocephalides* spp. and *Pulex* spp.) as well as lice (*Heterodoxus spiniger*, *Linognathus setosus*) as vectors [109]. The *Acanthocheilonema* species labelled *A. reconditum*-like in Figure 5 from domestic dogs in India was also demonstrated to develop to infective third larvae in *H. longipennis* [40]. The seal heartworm *A. spirocauda* is transmitted by the seal louse *Echinophthirius horridus* [110, 111]. It is interesting that adults of the latter species are not located in sites where they cause little damage, such as the peritoneal cavity (*A. dracunculoides*) or the subcutis (*A. reconditum*), but in the heart, where they can potentially cause anorexia and fatigue that may lead to more severe heart and lung complications and sometimes death [112]. Without any pathological evidence obtained in necropsies, it cannot be predicted whether the *Acanthocheilonema* species found in hyenas causes only low to moderate pathologies as those in domestic dogs, or severe clinical symptoms as *A. spirocauda* in seals.

Our finding that Anaplasmataceae and *B. lengau*-like piroplasms were present significantly more often in hyenas in Namibia than in Tanzania may indicate underlying regional differences between southern and eastern Africa, in terms of tick species diversity and climatic factors. Human activities are also likely to play a role through their effect on vector abundance and distribution, the density of hosts for larval stages and density of other competent host species that share pathogens with brown and spotted hyenas [113]. As expected, higher frequencies of some pathogens also resulted in higher frequencies of co-infection with multiple pathogens. Co-infections with multiple pathogens is generally the rule and not an exception in wildlife [114], but currently little is known of the effect concurrent infection with multiple pathogens has on wildlife at the individual or population level. Potential interactions include competition for resources such as host cells or nutrients, interaction via immune responses and production of chemical compounds. Pathogens may facilitate each other mutually, or only in a single direction, by suppressing the immune system, or might antagonise each other in cases where they compete for the same host cell or nutrients provided by the host. Indirect immunological interactions might lead to synergistic or antagonistic interactions between pathogens [114].

Considering that most emerging diseases are zoonotic and that most of these originate from wildlife, studies looking more systematically into co-infections in wildlife are urgently needed. The application of metagenomics methods in future studies on co-infections in wildlife should produce new insights [114], as was recently demonstrated by a study using faecal samples from SHTZ [115]. Additionally, as gastrointestinal helminth parasites can strongly interact with concurrent systemic malaria parasites located in very different organ systems [116, 117], analyses of multiple samples such as blood, faeces and urine from the same animal would improve knowledge on how co-infecting parasites interact and how their joint presence and activities affect their hosts.

## Conclusions

This study presents the relative frequencies of infections of several vector-borne pathogens in two hyena species from two African regions and three study sites with different ecological conditions. In general, the brown and spotted hyenas in Namibia had higher infection frequencies and higher species richness in terms of co-infections than the spotted hyenas in Tanzania. These differences are more likely related to differences in ecological conditions in geographic regions and study areas and the variable degree of vector-related human activities than to differences between the two species in terms of their immunological defences against infection. The genetic diversity of several parasite species was very high, suggesting that for several *Acanthocheilonema* spp., *Babesia* spp. and presumably also *Hepatozoon* spp. new species were detected that should be fully described with morphological details and deposition of type material in the future. Species not identical to well-described parasites of domestic dogs and domestic cats such as *A. dracunculoides*, *B. vogeli* and *H. canis* may have different life cycles and arthropod vectors that require parasitological and molecular investigation. The large number of *Babesia* species (including *B. lengau-like* genotypes) recently described from domestic cats [118] suggests that many of these pathogens may infect domestic cats and dogs and cause severe disease but have been overlooked so far [10, 86]. Currently, little is known regarding the pathology and outcome of infection caused by these pathogens in brown and spotted hyenas, and the same is true for co-infections that were common, particularly in Namibia. Further research is needed to assess the effect of these vector-borne pathogens on the health status and survival of brown and spotted hyenas.

## Supplementary Information

**Additional file 1: Table S1.** Raw data on all hyena specimens. Data include an arbitrary ID, the host species, an assigned group (BHNA, brown hyena Namibia, SHNA, spotted hyena Namibia; SHTZ, spotted hyena Tanzania), the year of collection, age (subadult, adult), the sample tissue, the area where the individual was found (farmland, Etosha NP, Serengeti NP), the country and region within the country. Results of PCRs for different pathogen groups are indicated as 0 and 1 for positive and negative, respectively. Sequencing results columns show the best taxonomic assignment to the sample based on sequencing data. Finally, the number of pathogen taxa detected in each specimen are indicated.

**Additional file 2: Table S2.** Primer sequences and PCR conditions.

## Data Availability

All data generated or analysed during this study are included in this article and its additional files. Sequence data were deposited in the GenBank database under the accession numbers listed in Additional file 1: Table S1.

## Abbreviations

BHNA: Brown hyena Namibia
SHNA: Spotted hyena Namibia
SHTZ: Spotted hyena Tanzania
NP: National park
*glt*A: Citrate synthase
rRNA: Ribosomal RNA
ITS1: Internal transcribed spacer 1
ITS2: Internal transcribed spacer 2
