# Supplementary material for: Genetic diversity of vector-borne pathogens in spotted and brown hyenas from Namibia and Tanzania relates to ecological conditions rather than host taxonomy
Source: Parasit Vectors. 2021 Jun 16;14:328. doi: 10.1186/s13071-021-04835-x (PMC8207800; doi:10.1186/s13071-021-04835-x)
Supplement: Supplementary file 2 — Additional file 2: Table S2. Primer sequences and PCR conditions. [file 13071_2021_4835_MOESM2_ESM.docx]

**Table S2**

PCR primer and conditions

| Target pathogen | Primer name | Referenz for primer | Primer sequence | Amplicon size (bp) | Enzyme | Denaturation/ Annealing temperature (°C) | Times for PCR steps (s)^a^ |
| --- | --- | --- | --- | --- | --- | --- | --- |
| Piroplasmida 18S rRNA | RLB-F2 | Matjila et al., 2004 | 5'-GAC ACA GGG AGG TAG TGA CAA G-3' | 460-540 bp | Phusion^a^ | 98/65 | 30/15/30/30/300 |
|  | RLB-R2 |  | 5'-CTA AGA ATT TCA CCT CTG ACA GT-3' |  |  |  |  |
|  | BlengauFor | new | 5'-GAA ATA ACA ATA CAG GGC TTT AAG C-3' |  | Phusion^a^ | 98/65 | 30/20/30/30/300 |
|  | RLB-R2 | Matjila et al., 2004 | 5'-CTA AGA ATT TCA CCT CTG ACA GT-3' |  |  |  |  |
| Onchocercidae ITS-2 | DIDR-F1 | Rishniw et al., 2006 | 5'-AGT GCG AAT TGC AGA CGC ATT GAG-3' | Highly variable, species dependent | Phusion^a^ | 98/60 | 30/10/30/30/300 |
|  | DIDR-R1 |  | 5'-AGC GGG TAA TCA CGA CTG AGT TGA-3' |  |  |  |  |
| Onchocercidae 12S rRNA | 12SF | Casiraghi et al. 2004 | 5'-GTTCCAGAATAATCGGCTA-3' | ~510 bp | Maxima^b^ | 94/52 | 60/30/45/60/600 |
|  | 12SRdeg |  | 5'-ATTGACGGATGRTTTGTACC-3' |  |  |  |  |
| *Rickettsia* sp. *gltA* | CS409d | Roux et al.,1997 | 5'-CCTATGGCTATTATGCTTGC-3' | 602-639 | Phusion^a^ | 98/60 | 20/10/30/30/300 |
|  | Rmasglta 1065lo | Schreiber et al., 2014 | 5'-TCAATAAAATATTCATCTTTAAGAGC-3' |  |  |  |  |
| Anaplasmataceae 16S rRNA | A/Efor | Tabar et al., 2008  modified by  Krücken et al., 2013 | 5'-GGG GAT GAT GTC AAR TCA GCA-3' | ~250 | Phusion^a^ | 94/60 | 300/30/30/30/360 |
|  | A/Erev |  | 5'-CAC CAG CTT CGA GTT AAG CCA AT-3' |  |  |  |  |
| *Hepatozoon* sp. 18S rRNA | HepF | Inokuma et al., 1992 | 5'-ATACATGAGCAAAATCTCAAC-3' | ~500 bp | Maxima^b^ | 94/56 | 240/15/30/40/600 |
|  | HepR |  | 5'-CTTATTATTCCATGCTGCAG-3' |  |  |  |  |

^a^Initial denaturation, denaturation/annealing/extension/final extension times

^b^Phusion Hot Start II High Fidelity DNA Polymerase

^c^Maxima Hot Start Taq DNA Polymerase
